# Force-Velocity Characteristics, Muscle Strength, and Flexibility in Female Recreational Marathon Runners

**DOI:** 10.3389/fphys.2018.01563

**Published:** 2018-11-02

**Authors:** Pantelis Theodoros Nikolaidis, Thomas Rosemann, Beat Knechtle

**Affiliations:** ^1^Exercise Physiology Laboratory, Nikaia, Greece; ^2^Laboratory of Exercise Testing, Hellenic Air Force Academy, Acharnes, Greece; ^3^Institute of Primary Care, University of Zurich, Zurich, Switzerland; ^4^Medbase St. Gallen Am Vadianplatz, St. Gallen, Switzerland

**Keywords:** aging, anthropometry, countermovement jump, cycle ergometer, handgrip strength, sit-and-reach test, squat jump

## Abstract

Physical fitness components that relate with performance in marathon running, e.g., aerobic capacity and body composition, have been studied extensively. On the other hand, data on components of the health-related physical fitness, such as flexibility and muscle strength, were missing in this sport. Therefore, the aim of the present study was to profile force-velocity (F-v) characteristics, muscle strength and flexibility in female recreational marathon runners and to examine their relationship with age, race time and anthropometric characteristics (body fat percentage, fat-free mass – FFM, and total thigh muscle cross-sectional area – CSA). Thirty three female marathon runners (age 40.0 ± 8.9 years, body fat percentage 19.5 ± 4.6% and personal record 4:34 ± 0:39 h:min), separated into three age groups (<35, 35–45 and >45 years) and three performance groups (race time <4:15 h:min, 4:15–4:45 h:min and >4:45 h:min), performed sit-and-reach test (SAR), isometric muscle strength tests, squat jump, countermovement jump and F-v test on a cycle ergometer. The main findings of the present study were that (i) participants had moderate scores of body composition and physical fitness considering norms of the general population, (ii) the <35 age group had better jumping ability than 35–45 and >45 age group, and the older age group had lower F_0_, Pmax and rPmax than their younger counterparts, (iii) the slowest performance group scored the highest in SAR, and (iv) isometric strength, F_0_ and Pmax correlated largely with body mass and FFM. Considering the lack of existing data on anaerobic power and neuromuscular fitness of female marathon runners, the findings reported in this study would be useful for strength and conditioning trainers to monitor the training of their athletes. Even if these parameters were not related to race time, they should be monitored regularly as they were either component of health-related physical fitness (muscle strength and flexibility) or could help runners (anaerobic power) under specific circumstances such as ascends during a race.

## Introduction

During the last decades, an increased number of female runners participate in marathon races. For instance, the male-to-female runners ratio of finishers in the “New York City Marathon” decreased from 2.1 in 2006 to 1.4 in 2016 (Nikolaidis et al., 2018). Accordingly, an increased scientific interest has evolved for female marathon runners. With regards to their physiological characteristics, most studies focused on maximal oxygen uptake (VO_2_max), anaerobic threshold and running economy (Helgerud et al., 1990; Daniels and Daniels, 1992; Pate and O’Neill, 2007). An explanation of the large number of studies focusing on these characteristics might be their correlations with endurance performance and marathon race time (Williams and Cavanagh, 1987; Midgley et al., 2007) and the role of aerobic capacity for health (Lee et al., 2010, 2011). On the other hand, anaerobic power or major components of health-related physical fitness, such as muscle strength and flexibility (Pate, 1983; Caspersen et al., 1985; Heyward and Gibson, 2014), have been rarely investigated in female marathon runners. Although some of the health-related physical fitness components (i.e., muscle strength and flexibility) might not be determinants of race time in marathon running, these components would allow humans performing daily physical activity with vigor (Cattuzzo et al., 2016). In addition, an optimal health-related physical fitness might play a key role for quality of life and successful aging (Lee and Tanaka, 1997).

Anaerobic power may be necessary in ascends or descends, or in situations such as to overcome rapidly an obstacle or an opponent during a marathon race, e.g., it has been shown that running in an augmented slope resulted in increased step frequency, ground reaction force and metabolic cost (Padulo et al., 2013). It has been shown that anaerobic power in elite marathon runners is relatively low compared with runners of shorter distances (Vuorimaa, 1996; Legaz-Arrese et al., 2011). For example, the results of a 20 s maximal anaerobic running test showed lower scores in elite marathon runners than sprinters and middle distance runners (Vuorimaa, 1996). Moreover, a comparative study of various running distances revealed that elite marathon runners had the lowest scores in the Wingate anaerobic test (WAnT) compared to their peers participating in shorter distances (Legaz-Arrese et al., 2011). So far, the force-velocity (F-v) test (Driss and Vandewalle, 2013), developed to characterize limits of the neuromuscular system to produce power (Cross et al., 2017), has been widely used in athletes such as judokas, boxers, taekwondo athletes (Busko, 2016), team handball (Nikolaidis et al., 2016), soccer (Nikolaïdis, 2012), tennis (Durand et al., 2010), and cyclists (Nikolaidis and Papadopoulos, 2011), but not in endurance athletes. Compared to the WAnT that evaluates anaerobic power, the F-v test provides additional information about the two constituents (i.e., force and velocity) of anaerobic power; thus, it can identify potential “weak” constituent that should be targeted for optimization (Driss and Vandewalle, 2013).

Few studies have even reported isometric muscle strength (e.g., handgrip) and jump ability (e.g., countermovement jump – CMJ) of female marathon runners in designs that used small number of female participants (*n* ≤ 6) (Del Coso et al., 2013; Piacentini et al., 2013). In addition, the abovementioned studies did not distinguish scores between sexes; thus, we have no knowledge about performance of female runners in these tests. CMJ has been shown to differentiate among male runners of various distances (e.g., marathon versus middle-distance versus sprinters) (Vuorimaa, 1996); thus, it could provide useful insight of practical relevance for female marathon runners. In addition, handgrip muscle strength is related to health; for instance, it has been shown that higher level of this physical fitness component is associated with a reduced risk of all-cause mortality, especially in women (Garcia-Hermoso et al., 2018).

With regards to flexibility, an optimal level of this parameter is necessary for health; for example, the flexibility of the muscle, tendons and ligaments in the back might be associated with the range of motion and functional movement (Gordon and Bloxham, 2016). A study on international level male distance runners showed that the least flexible runners were also the most economical in terms of running economy (Jones, 2002) indicating a concern that chronic endurance training could induce a decreased flexibility. However, these findings remain to be confirmed in female marathon runners. Although the abovementioned studies improved our understanding of the demands of marathon running for anaerobic power, muscle strength and flexibility in female runners, no information exists concerning relatively large samples of female recreational marathon runners, especially with regards to the variation of these characteristics with age, performance, and anthropometry. Such information would be of great practical value for strength and conditioning coaches working with female marathon runners to evaluate the fitness level of their athletes and develop specific training programs. Therefore, the main aim of the present study was to profile F-v characteristics, isometric muscle strength, jump ability, and flexibility of female recreational marathon runners and examine their relationship with age, performance and anthropometry.

## Materials and Methods

### Experimental Approach to the Problem

To examine the relationship of F-v characteristics, isometric muscle strength, jump ability and flexibility with age, performance and anthropometry, a cross-sectional study design was applied. Participants (*n* = 33) were divided into three age groups (<35, 35–45 and >45 years) to study the effect of age on these performance parameters. Also, differences among three even performance groups, based on the best race time (fast 3:53 ± 0:19 h:min, <4:15 h:min, *n* = 11; medium 4:30 ± 0:07 h:min, 4:15–4:45 h:min, *n* = 11; slow 5:14 ± 0:30 h:min, >4:45 h:min, *n* = 11), were examined. The best race time was considered instead of the most recent race time as the latter could be influenced by non-performance conditions, e.g., injury. The relationship of physical fitness (sit-and-reach test – SAR, squat jump – SJ, CMJ, isometric strength, and F-v test) with anthropometric characteristics (body mass, body mass index – BMI, body fat percentage – BF, total thigh muscle cross-sectional area – CSA, and fat-free mass – FFM) was examined using correlations.

### Subjects

Thirty-three female recreational marathon runners (age 40.0 ± 8.9 years, height 162 ± 6 cm, body mass 57.7 ± 7.4 kg, BMI 21.8 ± 2.1 kg.m^−2^ and personal record 4:34 ± 0:39 h:min, completed marathons in the past 3.3 ± 3.6) mostly from the area of Athens volunteered to participate in this study, which had been advertised through popular websites for endurance runners. The participants reported sport experience 5.6 ± 4.6 years and were practicing endurance training for 4.1 ± 1.5 days weekly with each training session corresponding to 9.3 ± 2.8 km or 1.6 ± 0.5 h. During September and October 2017, the participants visited the laboratory where they were examined for anthropometric characteristics and performed a F-v test, isometric muscle strength, jump ability and flexibility. This study was approved by the local Institutional Review Board (Exercise Physiology Laboratory, Nikaia). The study was conducted in accordance with the Declaration of Helsinki. All participants gave written informed consent after having been provided detailed information about the risks and benefits of the research.

### Procedures

#### Anthropometry

Height, body mass, and skinfolds were measured with participants in minimal clothing and barefoot. An electronic weighing scale (HD-351; Tanita, Arlington Heights, IL, United States) was employed for measurement of body mass (to the nearest 0.1 kg), a portable stadiometer (SECA Leicester, United Kingdom) for height (0.001 m), and a caliper (Harpenden, West Sussex, United Kingdom) for skinfolds (0.2 mm). BMI was calculated as the quotient of body mass (kg) to height squared (m^2^), and BF was estimated from the sum of 10 skinfolds, i.e., cheek, wattle, chest I, triceps, subscapular, abdominal, chest II, suprailiac, thigh, and calf (Parizkova, 1978). FFM in kg was calculated as “body mass - (body mass ^∗^ BF/100).” CSA was calculated as “(4.68 ^∗^ midthigh circumference in cm) – (2.09 × anterior thigh skinfold in mm) – 80.99” (Housh et al., 1995).

#### Sit-and-Reach Test

The sit-and-reach test (SAR) was used to assess low back and hamstring flexibility (Mayorga-Vega et al., 2014). It was performed on a box providing 15 cm advantage, i.e., the participants scores 15 cm when they reach their toes. Two trials were performed with 1 min break between trials and the best score was recorded to the nearest 0.5 cm. Intra-class correlation coefficient (ICC) of single measures was 0.981 (95% confidence intervals, CI, 0.975; 0.986).

#### Isometric Muscle Strength Tests

To evaluate isometric muscle strength, the sum of four tests (right and left handgrip test, back test, back-and-leg test) in absolute and relative to body mass values was used. The handgrip test was performed with participants standing and having their elbow flexed at approximately 90°. They were instructed to squeeze the handle of the handgrip dynamometer (Takei, Tokyo, Japan) as hard as possible for 5 s. Two trials were performed for each hand, with a 1 min rest between trials. The best trial was recorded for each hand (Heyward, 2010). ICC was 0.945 (95% CI, 0.926; 0.959) in the both hands. A back strength dynamometer (Takei, Tokyo, Japan) was used for both back strength test and back-and-leg test (test-retest ICC 0.92) (Ten Hoor et al., 2016). The back strength test was performed with participants having their legs and backs straightened to allow the bar to level with the patella, while in the combined back-and-leg test, the chain length on the dynamometer was adjusted so that the participants squatted over the dynamometer with their knees flexed at approximately 30° (Heyward, 2010). All measurements were recorded to the nearest 0.1 kg.

#### Jumping Tests

The participants performed two trials for each jumping test (squat jump, SJ, and countermovement jump, CMJ) and the best result was recorded (Aragon-Vargas, 2000). There was 1 min break between trials and tests. Height of each jump was estimated using the Opto-jump (Microgate Engineering, Bolzano, Italy) and was expressed to the nearest 0.1 cm. ICC was 0.914 (95% CI, 0.885; 0.936) in SJ and 0.951 (95% CI, 0.934; 0.963) in CMJ.

#### Force-Velocity Test

The F-v test was used to assess Pmax, expressed as W and as W⋅kg^−1^ (rPmax), theoretical maximal velocity (v_0_) in revolutions per minute (rpm) and force (F_0_) in N, and v_0_/F_0_ was calculated in rpm.N^−1^. The participants performed four sprints, each one lasting 7 s, against braking force (2, 3, 4, and 5 kg on a counterbalanced order) on a leg cycle ergometer (Ergomedics 874E, Monark, Sweden), interspersed by 5 min recovery periods. The seat height of the ergometer was adjusted to allow for a slight bend in the knee (approximately 175°) and in accordance with the participant’s satisfaction. Each sprint began with a flying start, i.e., as soon as velocity reached 50 rpm (revolutions per minute), the weight basket was released and the braking force was applied. For each participant an individual linear regression was determined between peak velocity and braking force for each of the four sprints. F_0_ and v_0_ corresponded to the intercepts with F and v axes in the F-v graph. Pmax was calculated as Pmax = 0.25⋅F_0_⋅v_0_ (Vandewalle et al., 1985).

### Statistical Analyses

Statistical analyses were performed using IBM SPSS v.20.0 (SPSS, Chicago, IL, United States) and GraphPad Prism v. 7.0 (GraphPad Software, San Diego, CA, United States). Normality was examined using Kolmogorov-Smirnov test and visual inspection of normal Q-Q plots. Data were expressed as mean and standard deviation (SD). One-way repeated measures analysis of variance (ANOVA) and a subsequent Bonferroni *post hoc* test (if there were differences among groups) were used to examine the differences among age and performance groups, separately. To interpret effect size (ES) for statistical differences in the ANOVA, partial eta square classified as small (0.010 < η*_p_*^2^≤ 0.059), medium (0.059 < η*_p_*^2^≤ 0.138), and large (η*_p_*^2^ > 0.138) was used (Cohen, 1988). The relationship of flexibility, isometric muscle strength, jumping ability and F-v characteristics with age, performance and anthropometry was examined using Pearson’s product moment correlation coefficient (*r*). Magnitude of correlation coefficients was considered as trivial if *r* < 0.10, small if 0.10 ≤*r* < 0.30, moderate if 0.30 ≤*r* < 0.50, large if 0.50 ≤*r* < 0.70, very large if 0.70 ≤*r* < 0.90, nearly perfect if *r* ≥ 0.90, and perfect if *r* = 1.00 (Batterham and Hopkins, 2006). The level of significance was set at α = 0.05.

## Results

### Profile

The anthropometric characteristics of participants, in total and by age group, can be seen in Table 1. In the F-v test, v_0_ of all participants was 167 ± 15 rpm (ranging from 132 to 195 rpm), F_0_ 120 ± 20N (93–179 N), Pmax 507 ± 85W (340–704 W), rPmax 8.83 ± 1.17 W.kg^−1^ (6.0–11.1 W.kg^−1^) and v_0_.F_0_^−1^ 1.43 ± 0.28 rpm.N^−1^ (0.87–1.93 rpm.N^−1^). With regards to neuromuscular fitness, SAR was 25.8 ± 8.3 cm (8.0–37.5 cm), SJ 17.7 ± 3.4 cm (10.3–25.2 cm) and CMJ 18.6 ± 3.7 cm (11.2–26.1 cm). In isometric muscle strength, right handgrip was 29.7 ± 4.5 kg (20.7–41.6 kg), left handgrip 29.7 ± 4.0 kg (23.4–39.7 kg), back 82.5 ± 16.2 kg (40.0–114.0 kg), back-and-leg 94.9 ± 19.0 kg (48.5–150.0 kg), sum 236.9 ± 40.1 kg (134.2–339.3 kg) and 4.12 ± 0.56 kg.kg^−1^ (2.90–5.23 kg.kg^−1^).

**Table 1:** Comparison of anthropometric characteristics among age groups.

	Total	Age groups		
		<35 years	35–45 years	>45 years
n	33	10	13	10
Age (years)^∗^	40.0 ± 8.9	29.2 ± 4.5	41.0 ± 2.2	49.3 ± 5.3
Height (cm)	162 ± 6	161 ± 6	163 ± 6	163 ± 7
Body mass (kg)	57.7 ± 7.4	53.7 ± 6.9	58.5 ± 6.5	60.5 ± 7.8
BMI (kg.m^−2^)	21.8 ± 2.1	20.7 ± 1.6	22.0 ± 1.6	22.7 ± 2.8
BF (%)	19.5 ± 4.6	17.3 ± 5.0	19.5 ± 3.5	21.8 ± 4.9
FFM (kg)	46.3 ± 5.2	44.4 ± 6.0	47.0 ± 4.3	47.2 ± 5.3
CSA (cm^2^)	118 ± 17	119 ± 14	120 ± 16	114 ± 21

*BMI, body mass index; BF, body fat percentage; FFM, fat-free mass; CSA, thigh muscle cross-sectional area. ^∗^Except age for which all age groups differed at p < 0.001, no difference was observed in any anthropometric characteristic.*

### Age

**FIGURE 1:**
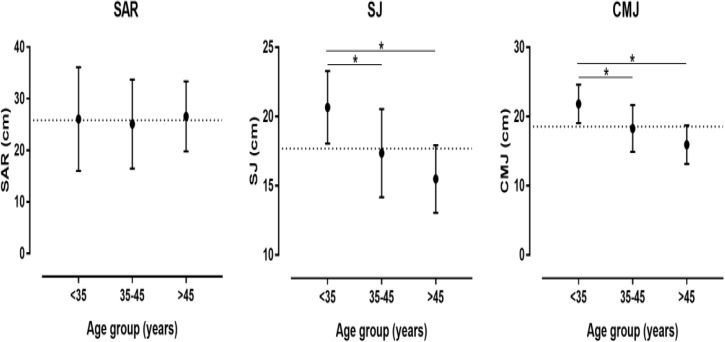
Differences in sit-and-reach test (SAR), squat jump (SJ) and countermovement jump (CMJ) among age groups. **^∗^** Difference at *p* < 0.05.

**FIGURE 2:**
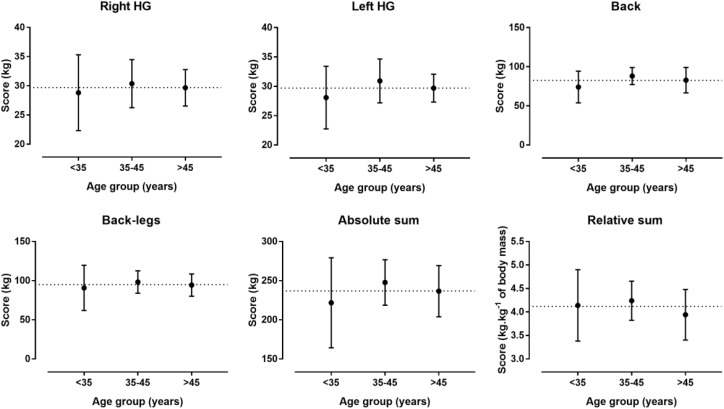
Differences in isometric muscle strength among age groups. **^∗^** Difference at *p* < 0.05.

**FIGURE 3:**
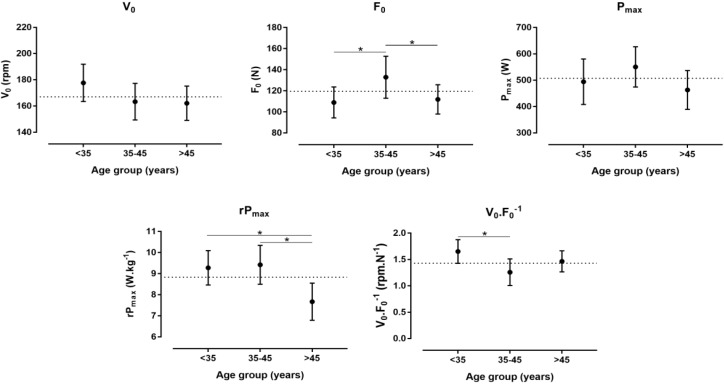
Differences in theoretical maximal velocity (v_0_), force (F_0_), maximal power in absolute (Pmax) and relative values (rPmax), and v_0_.F_0_^-1^ among age groups. **^∗^** Difference at *p* < 0.05.

A large main effect of age on SJ (*p* = 0.002, η*_p_*^2^ = 0.361) and CMJ (*p* = 0.001, η*_p_*^2^ = 0.384) was observed with higher values in the <35 age group than in the 35–45 and >45 age group, whereas no difference was shown in SAR (*p* = 0.912, η*_p_*^2^ = 0.006) (Figure 1). No difference was found in isometric muscle strength (Figure 2). With regards to F-v characteristics, a large main effect was observed on v_0_ (*p* = 0.034, η*_p_*^2^ = 0.208), F_0_ (*p* = 0.004, η*_p_*^2^ = 0.323) with higher score in 35–45 than <35 and >45 age group, Pmax (*p* = 0.037, η*_p_*^2^ = 0.203) with higher score in 35–45 than >45 age group, rPmax (*p* < 0.001, η*_p_*^2^ = 0.468) with higher score in <35 and 35–45 than >45 age group and v_0_/F_0_ (*p* = 0.002, η*_p_*^2^ = 0.354) with higher score in <35 than 35–45 age group (Figure 3).

### Anthropometry and Physical Fitness by Performance Group

Anthropometric characteristics and physical fitness by performance level can be seen in Table 2. A large main effect of performance on flexibility was observed (*p* = 0.039, η*_p_*^2^ = 0.201) with higher score (+8.3 cm) in the slowest than in the average performance group.

**Table 2:** Comparison among performance groups (quartiles).

	Performance groups		
	Fast (*n* = 11)	Average (*n* = 11)	Slow (*n* = 11)
Age (years)	38.8 ± 10.7	41.0 ± 7.8	40.0 ± 8.6
Height (cm)	163 ± 7	162 ± 5	162 ± 8
Body mass (kg)	58.1 ± 7.4	57.6 ± 6.6	57.3 ± 8.6
BMI (kg.m^−2^)	21.7 ± 1.7	21.8 ± 1.9	21.9 ± 2.9
BF (%)	19.3 ± 2.6	20.6 ± 3.9	18.7 ± 6.6
FFM (kg)	47.0 ± 6.5	45.5 ± 4.1	46.3 ± 5.0
CSA (cm^2^)	120 ± 18	117 ± 14	118 ± 20
SAR (cm)	24.0 ± 8.1	22.5 ± 7.6^∗^	30.8 ± 7.2^∗^
SJ (cm)	18.6 ± 3.5	17.6 ± 3.3	17.0 ± 3.5
CMJ (cm)	19.0 ± 3.9	18.7 ± 3.9	18.0 ± 3.8
**Isometric muscle strength**			
Right HG (kg)	30.8 ± 6.2	30.5 ± 4.0	28.0 ± 2.9
Left HG (kg)	30.8 ± 4.6	30.6 ± 4.0	28.0 ± 2.9
Back (kg)	89.8 ± 20.7	83.4 ± 10.0	75.1 ± 14.4
Back-legs (kg)	104.9 ± 26.4	94.4 ± 12.7	86.5 ± 12.6
Absolute sum (kg)	256.2 ± 54.4	238.8 ± 23.8	217.5 ± 30.8
Relative sum (kg.kg^−1^)	4.39 ± 0.69	4.16 ± 0.24	3.84 ± 0.58
**Force-velocity test**			
v_0_ (rpm)	167 ± 20	170 ± 13	165 ± 12
F_0_ (N)	118 ± 22	121 ± 24	120 ± 14
P_max_ (W)	503 ± 114	517 ± 81	502 ± 63
rP_max_ (W.kg^−1^)	8.59 ± 1.17	9.04 ± 1.34	8.84 ± 1.04
v_0_.F_0_^−1^ (rpm.N^−1^)	1.45 ± 0.30	1.46 ± 0.30	1.39 ± 0.24

*BMI, body mass index; BF, body fat percentage; FFM, fat-free mass; CSA, thigh muscle cross-sectional area; SAR, sit-and-reach test; SJ, squat jump; CMJ, countermovement jump; HG, handgrip; v_0_, theoretical maximal velocity; F_0_, force; P_max_, maximal power in absolute values; rP_max_, maximal power in relative values. ^∗^Difference at p = 0.05.*

### Anthropometry

SJ and the sum of isometric strength tests (in relative values) correlated inversely moderately with BF (Table 3). All the other indices of isometric strength correlated largely with body mass, FFM and CSA. It should be highlighted that the magnitude of these correlations with FFM was larger than body mass. Vo and rPmax did not correlate with any anthropometric characteristic, whereas F0 and Pmax correlated largely with body mass and FFM.

**Table 3:** Correlations between anthropometric characteristics and physical fitness tests.

	Body mass	BMI	BF	CSA	FFM
SAR	0.149	0.067	−0.302	0.195	0.316
SJ	−0.112	−0.202	−0.418^∗^	−0.027	0.078
CMJ	−0.123	−0.160	−0.301	−0.027	0.007
**Isometric muscle strength**					
Right HG	0.674^‡^	0.345	0.103	0.430^∗^	0.710^‡^
Left HG	0.699^‡^	0.460^†^	0.140	0.500^†^	0.719^‡^
Back	0.487^†^	0.221	−0.016	0.460^†^	0.562^†^
Back-legs	0.514^†^	0.220	−0.056	0.484^†^	0.622^‡^
Absolute sum	0.587^‡^	0.278	−0.008	0.514^†^	0.674^‡^
Relative sum	−0.159	−0.347	−0.401^∗^	0.158	0.034
**Force-velocity test**					
v_0_	0.064	−0.023	−0.069	0.121	0.111
F_0_	0.562^†^	0.435^∗^	0.130	0.345	0.563^†^
P_max_	0.604^‡^	0.416^∗^	0.091	0.407^∗^	0.633^‡^
rP_max_	−0.207	−0.219	−0.309	0.007	−0.078
v_0_.F_0_^−1^	−0.451^†^	−0.352^∗^	−0.102	−0.261	−0.450^∗^

*BMI, body mass index; BF, body fat percentage; FFM, fat-free mass; CSA, thigh muscle cross-sectional area; SAR, sit-and-reach test; SJ, squat jump; CMJ, countermovement jump; HG, handgrip; v_0_, theoretical maximal velocity; F_0_, force; P_max_, maximal power in absolute values; rP_max_, maximal power in relative values. ^∗^p < 0.05, ^†^p < 0.01, ^‡^p < 0.001.*

## Discussion

The main findings of the present study were that (i) participants had average scores of body composition and physical fitness compared to the general population, (ii) the <35 age group had better jumping ability than 35–45 and >45 age group, and the older age group had lower F_0_, Pmax and rPmax than their younger counterparts, (iii) the slowest performance group scored the highest in SAR, and (iv) isometric strength, F0 and Pmax correlated largely with body mass and FFM.

### Profile

CMJ was in similar levels as sex- and age-matched general population (Edwen et al., 2014). Participants had lower CMJ and F-v characteristics than volleyball players (Nikolaidis, 2013) indicating that female marathon runners characterized by physiological range of muscle strength and power. This observation was in agreement with research in male runners, where relatively low scores of anaerobic power were shown in marathon runners compared to shorter distances’ runners (Vuorimaa, 1996; Legaz-Arrese et al., 2011). For instance, CMJ in male marathon runners was ∼13 cm and 24 cm lower than middle-distance runners and sprinters, respectively (Vuorimaa, 1996). All measures of isometric muscle strength (right and left handgrip, back, leg, total, and relative) were classified as average compared to general population (Heyward and Gibson, 2014).

### Age

The older age group had lower jumping ability, F_0_, Pmax and rPmax than their younger counterparts, suggesting a decline of these physical fitness components with aging and indicating that probably the chronic endurance exercise does not prevent from loss in muscle strength and power. It should be highlighted that the participants had sport experience in endurance running for 5.6 years. These findings confirmed previous studies in male participants which showed a decline of anaerobic power with aging (Chamari et al., 1995; Bonnefoy et al., 1998). For instance, in a comparison between young (25 years) and master athletes (65 years) matched for weight, height and training, P_max_ was ∼43%, F_0_ 30% and v_0_ 15% lower in the older athletes (Chamari et al., 1995). A study of young (23 years) and elder male participants (71 years) showed a decline of rP_max_ by 8% per decade and of velocity by 4%, and a moderate inverse relationship between rP_max_ and age (*r* = -0.33) (Bonnefoy et al., 1998).

### Performance

Among all anthropometric characteristics and physical fitness components examined in the present study, flexibility was the only one observed to differ among performance groups with the slowest one presenting the better score in SAR than the average group. This observation might be due to that running economy (which is related with performance) is inversely correlated with sit-and-reach test score (Jones, 2002; Trehearn and Buresh, 2009). An increased storage and return of elastic energy in stiffer musculotendinous structures might reduce the aerobic demand of submaximal running (Drew et al., 2011). In addition, no difference was shown among performance groups with regards to muscle strength and power. This finding was in agreement with a previous research showing that fast marathon runners were not characterized by high anaerobic power (Vuorimaa, 1996; Legaz-Arrese et al., 2011).

### Anthropometry

Most indices of muscle strength and power correlated with both body mass, FFM and CSA, which might explain why female marathon runners are not characterized by high levels of muscle strength and power as their body dimensions are relatively small compared to other sports. An excess of FFM, even if this is “active mass,” is a load that marathon runners need to carry with them; thus, their profile consists of small anthropometric characteristics and corresponding moderate levels of muscle strength and power. Although an increased Pmax might improved the cost of running (Giovanelli et al., 2017), its association with increased FFM would lead to slower race time.

### Limitations, Strength, Practical Applications

A limitation of the present study was the specificity of protocols that assessed the various physical fitness components as caution would be needed to compare their findings with studies using other protocols. For instance, F-v test and WAnT reflect different aspects of anaerobic power and capacity and their findings should not be used interchangeably (Jaafar et al., 2016). Strength of this study was its novelty since it was the first to examine flexibility, isometric muscle strength, two jump tests (SJ and CMJ) and F-v characteristics of female marathon runners and the findings could be used as norms and references for future studies. Considering the gap in the existing literature about these physical fitness components in female marathon runners, the findings add new information. The present study confirmed on female runners the findings of previous studies on male runners showing that flexibility, muscle strength and power were not related to performance in marathon runners. On the other hand, flexibility and muscle strength are components of the health-related physical fitness, and in this context, they should be regularly monitored in addition to sport-related physical fitness components, such as aerobic capacity (maximal oxygen uptake, anaerobic threshold, and running economy). In view of the increased female participation in marathon races during the last decades (Lepers and Cattagni, 2012), the findings were of great practical value for strength and conditioning coaches in the context of training and testing of their runners. Surprisingly, although an optimal level of flexibility and muscle strength is important for health, these fitness components have been rarely studied in male marathon runners (Maud et al., 1981). Thus, the present study filled a gap in the existing literature as it was the first study – to the best of our knowledge – to provide data on F-v characteristics and the abovementioned health-related physical fitness components in female marathon runners.

## Conclusion

Profiling physical fitness characteristics of marathon runners is of great practical importance for strength and conditioning coaches working in this sport. The assessment of physical fitness assists to evaluate the effectiveness of training. So far, a lack of reference data on female marathon runners’ physical fitness, especially with regards to anaerobic power, muscle strength and flexibility, has been observed. Therefore, the data reported in this study would be useful for strength and conditioning trainers to monitor the training of female marathon runners. Strength and conditioning coaches may work with female marathon runners differing for age, performance level and anthropometric characteristics; thus, knowledge about the effect of age, performance and anthropometry on physical fitness would assist to accurately evaluate and prescribe training program. The findings highlighted the lower leg strength in the older age group; thus, strength and conditioning coaches should focus on the development of age-tailored training programs to enhance the jump ability of older female runners. On the other hand, flexibility should be monitored regularly targeting and be within a physiological range, whereas a high flexibility should be alarming as it associates with reduced performance in marathon. Considering the increased number of female finishers in marathon races during the last years, the findings have practical applications to a large number of recreational marathon runners. It should be also highlighted that the age and performance level of participants in the present study (∼40 years old and 4:34 h:min, respectively) was close to the average of finishers in large marathon races such as the “New York City marathon” (∼39 years old and 4:48 h:min, respectively).

## Author Contributions

PN performed the laboratory tests, analyzed the data and drafted the manuscript, TR and BK helped in drafting the manuscript.

## Conflict of Interest Statement

The authors declare that the research was conducted in the absence of any commercial or financial relationships that could be construed as a potential conflict of interest.
